# Genetic Diversity of a Natural Population of *Akebia trifoliata* (Thunb.) Koidz and Extraction of a Core Collection Using Simple Sequence Repeat Markers

**DOI:** 10.3389/fgene.2021.716498

**Published:** 2021-08-31

**Authors:** Yicheng Zhong, Yue Wang, Zhimin Sun, Juan Niu, Yaliang Shi, Kunyong Huang, Jing Chen, Jianhua Chen, Mingbao Luan

**Affiliations:** Institute of Bast Fiber Crops, Chinese Academy of Agricultural Sciences/Key Laboratory of Stem-Fiber Biomass and Engineering Microbiology, Ministry of Agriculture, Changsha, China

**Keywords:** *Akebia trifoliata* (Thunb.) Koidz, genetic diversity, simple sequence repeat markers (SSRs), genetic structure, core collection

## Abstract

Understand genetic diversity and genetic structure of germplasm is premise of germplasm conservation and utilization. And core collection can reduce the cost and difficulty of germplasm conservation. *Akebia trifoliata* (Thunb.) Koidz is an important medicinal, fruit and oil crop, particularly in China. In this study, 28 simple sequence repeat (SSR) markers were used to assess the genetic diversity and genetic structure of 955 *A. trifoliata* germplasms, determine their molecular identity and extract a core collection. The genetic diversity of the 955 germplasms was moderately polymorphic. The average number of alleles (Na), observed heterozygosity (H_*O*_), expected heterozygosity (H_*E*_), Shannon’s information index (I^∗^), and polymorphic information content (PIC) were 3.71, 0.24, 0.46, 0.81, and 0.41, respectively. Four subpopulations were identified, indicating a weak genetic structure. A 955 germplasms could be completely distinguished by the characters of s28, s25, s74, s89, s68, s30, s13, s100, s72, s77, and s3. And each germplasm’s molecular identity was made up of eleven characters. The core collection was composed of 164 germplasms (17.2% of 955 total germplasms in the population) and diversity parameters differed significantly from those of a random core collection. These results have implications for germplasm conservation. At the same time, based on the results, the 955 germplasm could be better used and managed.

## Introduction

*Akebia trifoliata* (Thunb.) Koidz belongs to the family *Lardizabalaceae* and genus *Akebia* Decne and is mainly distributed throughout China, Japan, North Korea, and Russia. In China, this species is mainly distributed in the Yangtze and Yellow River Basins, and Shaanxi-Sichuan area (Xie et al., 2006). *A. trifoliata* has a long history of practical use in traditional Chinese medicine (Xu et al., 2016). And in recent years, it has been developed as a fruit and oil crop (Zhao et al., 2014; Fang et al., 2021). Although *A. trifoliata* has a wide range of uses, the research progress on its biology has been slow, especially its domestication and genetics. At present, there is few work on the evolution, origin, domestication and so on of this species, which greatly limits its development. With the analysis of the genetic mechanism of main phenotypic characterization and its genome, it is expected to change the *status quo* (Niu et al., 2020; Huang et al., 2021).

*Akebia trifoliata* has different phenotypic characterization which needs to be improved for different purposes. For example, as a fruit, it needs to reduce the number of seeds; as a medicinal material, it needs to increase the content of medicinal ingredients; as an oil crop, it needs to increase the content of unsaturated fatty acids (Zhou et al., 2021). Genetic diversity and population structure is important for breeding of *A. trifoliata.* There are many different ways to determine genetic diversity and genetic structure, such as morphological, physiological, ecological characteristics, chromosome structure, biochemical and DNA marker (Rana et al., 2015). DNA marker is not influenced by growth and environmental conditions, so it is widely used in plant science (Agarwal et al., 2008). Among different DNA marker, simple sequence repeats (SSRs) are widely used, owing to their high polymorphism, reliability, rapid and simple detection, low cost, and easy operation. However, to the best of our knowledge, there have been few reports on *A. trifoliata* genetic diversity based on DNA markers. In previous study, the number of germplasm was few and only came from a part of China, so the results may not be representative (Niu et al., 2019; Zhang et al., 2021).

Germplasm conservation is essential to biodiversity and plant breeding, but it takes a lot of time and energy to collect and conserve as more germplasm as possible. However, redundant genetic resources present a challenge for the effective conservation, management, evaluation, and utilization of germplasm. To resolve this issue, it is necessary to construct a core collection to effectively preserve and utilize these germplasms (Holbrook and Anderson, 1995). Core collection were first proposed by Frankel, which provides preliminary information on diversity in a large collection (Frankel, 1984). Most core collections include only 5–20% of the total germplasms but preserve most of the genetic diversity, thereby reducing the cost and increasing the speed of the work process. Core collections have been established for many crops, even some important crops have established a variety of core collection, such as *rice* (Abadie et al., 2005; Yan et al., 2007), *wheat* (Balfourier et al., 2007; Kobayashi et al., 2016) and *maize* (Yu et al., 2005; Yan et al., 2009). However, thus far, only one core collection has been developed for *A. trifoliata*, and the germplasms were just collected from one province of China, therefore, the core collection had limitations (Zhang et al., 2021).

In the present study, 28 pairs of SSR markers were used to analyze the variation of 955 *A. trifoliata* germplasms which were collected from China. Whose aims are the followings: (1) to evaluate the overall genetic diversity and genetic structure (2) to establish molecular identity to distinguish 955 germplasms and (3) to construct a core collection to facilitate later management. Genetic diversity and genetic structure will be valuable to guide the available collection and application of *A. trifoliata* in China. Molecular identity and core collection will be beneficial to the *A. trifoliata* germplasm management and breeding.

## Materials and Methods

### Plant Materials and DNA Isolation

The 955 *A. trifoliata* germplasms, for which the collection location was unclear, were cultivated in the Taojiang experimental field of the Institute of Bast Fiber Crops, Chinese Academy of Agricultural Sciences in 2012. Fresh tender leaves from each germplasm were placed in a liquid nitrogen tank, transported to the laboratory, and frozen at −80°C until genomic DNA extraction. Genomic DNA was extracted using a Rapid DNA Extraction Kit (Tiangen Biotech, Beijing, China). The purity and quality of extracted DNA were evaluated by 1% agarose gel electrophoresis and determined using a NanoDrop 2000 spectrophotometer.

### SSR Analysis

From the SSR primers developed by Niu et al. (2019), 28 pairs of primers with high polymorphism were selected (Supplementary Table 1). SSR-primed polymerase chain reactions (PCRs) were carried out in a 10-μL reaction volume with 1 × PCR buffer, 0.2 mmol/L dNTP, 1 U of Taq DNA polymerase (Tiangen), 0.5 μL of forward primer (10 nmol/L), 0.5 μL of reverse primer (10 nmol/L), and 0.5 μL of DNA from each accession. PCR was performed under the following conditions: 94°C for 5 min, followed by 33 cycles each of 30 s at 95°C, 30 s at the primer-specific annealing temperature, 30 s at 72°C, and a final extension of 10 min at 72°C. The PCR products were separated on 8% polyacrylamide gels, and silver dyeing was conducted according to the methods of Zhang et al. (2000). Based on Ni et al. (2018), molecular weights were estimated using a DNA marker. The allele with the maximal molecular weight was recorded as “A,” followed by B, C, D. If only one band was obtained for a set of primers, the accession was recorded as homozygous.

### Establishment of Molecular Identity

1, 2, 3, 4 and A, B, C, D, E, F was used to instead of A/A, B/B, C/C, and D/D and A/B, A/C, A/D, B/C, B/D, and C/D genotype, respectively, and 0 represented no band. The character of the SSR marker which has the highest PIC was first to be used distinguish 955 germplasms. Then, the second, the third was added until all germplasm were completely distinguished. Finally, the molecular identity of each germplasm consisted of characters corresponding to the SSR markers.

### Extraction of a Core Collection

The stepwise clustering method can effectively preserve the genetic diversity of the original germplasm (Wang et al., 2007). Accordingly, in this study, stepwise clustering was used to extract a core collection based on SSR markers. First, genetic distances were calculated for the original collection, and a cluster analysis was then performed according to the genetic distances. Next, a tree diagram was obtained. According to the principle of clustering, the differences within groups were the smallest at the lowest level. Therefore, one of the two genetic materials in each group was randomly selected to enter the next round of the cluster analysis. If only one genetic material was available, then it was used in the next round of the cluster analysis. All retained genetic materials were re-entered into the next round of the cluster analysis. The method was repeated until the material met the set requirements to obtain the core collection.

### Data Analysis

PopGene version 1.3.2 was used to analyze the effective number of alleles (*N*_*e*_), Shannon–Weaver diversity index (*I*^∗^), genetic distance (Nei’s genetic distance), observed heterozygosity (*H*_*O*_), and expected heterozygosity (*H*_*E*_) (Yeh and Boyle, 1997); PowerMarker version 3.2.5 was used to estimate the polymorphic information content (PIC) and number of alleles (*N*_*a*_) (Liu and Muse, 2005). Based on Nei’s genetic distances and the unweighted pair group method with arithmetic mean (UPGMA), a clustering tree was constructed using PowerMarker and visualized using MEGA version 7.0 and iTol (Tamura et al., 2013). Population genetic structure was assessed using the mixed and correlated allele frequency models in STRUCTURE version 2.3.4 and Structure Harvester version 6.0 (Pritchard et al., 2000; Earl and Vonholdt, 2012). The variance analysis was implemented in SAS version 9.0 (Park, 2002).

## Results

### Genetic Diversity of 955 Germplasms

A total of 104 alleles were detected using 28 SSR markers. As summarized in Table 1, *N*_*a*_ per locus ranged from 2 to 5 (mean, 3.7143). Seventeen primer pairs amplified four alleles, and five primer pairs amplified two alleles, only one amplified five alleles. *N*_*E*_ ranged from 1.2018 to 2.8556 (mean, 1.9873), *H*_*O*_ from 0 to 0.83 (mean, 0.2382), *H*_*E*_ from 0.1681 to 0.6521 (mean, 0.4604), Nei’s distance from 0.1679 to 0.6535 (mean, 0.4600), *I*^∗^ from 0.3083 to 1.1866 (mean, 0.8086), and PIC from 0.1538 to 0.5936 (mean, 0.4085). The PIC indicated that the 28 SSR markers were moderately polymorphic (0.25 < PIC < 0.5). The most polymorphic SSR marker had 3.86 times higher variance than that of the least polymorphic marker. Seven microsatellites exhibited high polymorphism (PIC > 0.5), and four microsatellites exhibited low polymorphism (PIC < 0.25). The heterozygosity of *A. trifoliata* was relatively low based on *H*_*O*_ and *H*_*E*_ (i.e., 0.238 and 0.460, on average, respectively).

**TABLE 1:** Genetic diversity parameters for original genetic population at the 28 SSR markers.

**Marker**	**Na**	**Ne**	**Ho**	**He**	**Nei’s**	I*	**PIC**
s3	4.0000	2.1867	0.8331	0.5430	0.5427	0.8772	0.4518
s4	3.0000	1.2635	0.0000	0.2087	0.2085	0.3772	0.1881
s5	2.0000	1.2018	0.0000	0.1681	0.1679	0.3083	0.1538
s13	4.0000	2.4260	0.3389	0.5881	0.5878	0.9884	0.5184
s19	3.0000	1.2717	0.0597	0.2137	0.2136	0.4423	0.2029
s22	4.0000	1.9081	0.3488	0.4762	0.4759	0.8338	0.4180
s24	4.0000	1.4441	0.2630	0.3077	0.3075	0.5870	0.2857
s25	5.0000	2.8864	0.4837	0.6539	0.6535	1.1603	0.5917
s27	4.0000	1.5906	0.1328	0.3715	0.3713	0.6942	0.3412
s28	4.0000	2.8708	0.4882	0.6521	0.6517	1.1866	0.5936
s30	4.0000	2.6522	0.3899	0.6233	0.6230	1.1080	0.5553
s32	3.0000	1.3067	0.1883	0.2349	0.2347	0.4760	0.2217
s34	4.0000	1.6833	0.2664	0.4062	0.4059	0.7404	0.3665
s40	3.0000	2.0286	0.2000	0.5079	0.5071	0.8584	0.4434
s46	4.0000	1.6737	0.2225	0.4028	0.4025	0.7544	0.3686
s50	3.0000	1.5539	0.0881	0.3567	0.3565	0.5908	0.3030
s52	4.0000	1.9707	0.2440	0.4928	0.4926	0.8646	0.4415
s57	4.0000	1.6181	0.3394	0.3822	0.3820	0.6975	0.3494
s59	4.0000	1.5182	0.1046	0.3416	0.3423	0.6152	0.3037
s67	4.0000	2.0343	0.0986	0.5087	0.5084	0.8766	0.4509
s68	4.0000	2.6354	0.2705	0.6210	0.6206	1.0916	0.5578
s72	3.0000	2.2823	0.2358	0.5624	0.5619	0.9275	0.4834
s74	4.0000	2.8566	0.2374	0.6504	0.6499	1.1549	0.5879
s77	3.0000	2.3400	0.0400	0.5746	0.5726	0.9219	0.4787
s84	4.0000	1.6493	0.1595	0.3940	0.3937	0.7671	0.3654
s89	4.0000	2.6996	0.2158	0.6307	0.6296	1.0835	0.5599
s92	4.0000	1.9091	0.0346	0.4764	0.4762	0.7087	0.3712
s100	4.0000	2.1819	0.3846	0.5420	0.5417	0.9489	0.4854
Mean	3.7143	1.9873	0.2382	0.4604	0.4600	0.8086	0.4085

Marker, the name of SSR marker; Na, number of alleles; Ne, effective number of alleles; Ho, observed heterozygosity; He, expected heterozygosity; Nei’s, genetic distance; I*, Shannon’s information index; PIC, polymorphic information content.

### Genetic Structure of 955 Germplasms

**FIGURE 1:**
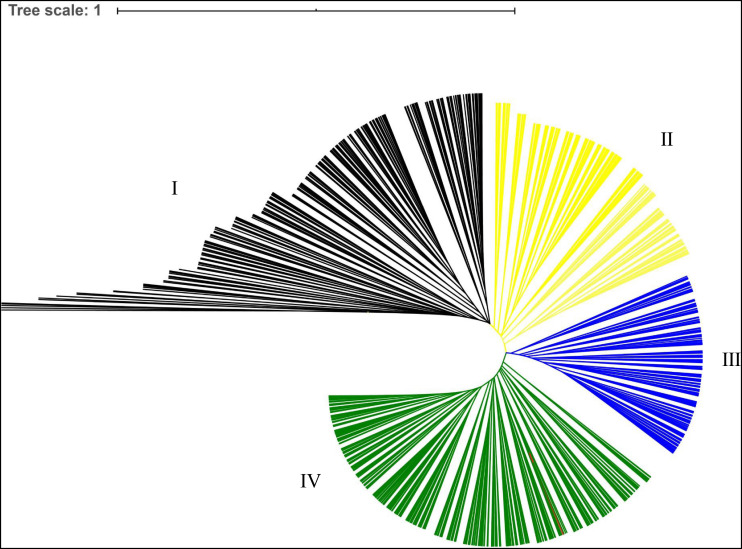
Phylogenetic tree of the 955 *A. trifoliate* accessions based on genetic distance. Blue and orange indicate different clusters.

A cluster analysis was performed to analyze the genetic relationships among the 955 *A. trifoliata* accessions, and a dendrogram based on genetic distances and the unweighted pair group method with arithmetic mean is shown in Figure 1. The cluster analysis divided 955 germplasms into four main groups, accounting for 25.03, 18.32, 17.07, and 39.58% of the natural population, respectively.

**FIGURE 2:**
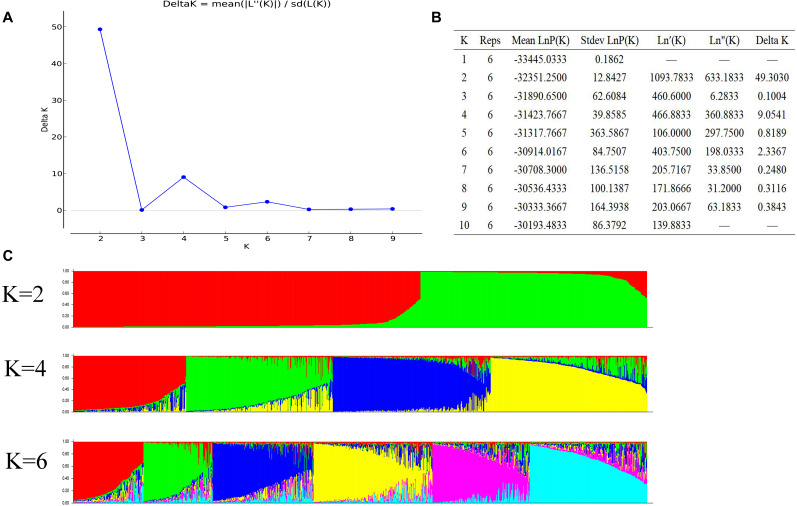
Population structure analysis of the 955 *A. trifoliate* accessions. **(A)** Delta K based on the rate of change of L (K) between successive *K*-values. **(B)** Evanno table output. **(C)** Population structure based on *K* = 2, 4, 6.

We evaluated *K-values* (population number) of 1 to 10 for a STRUCTURE analysis. The most significant change in likelihood occurred when the *K-value* increased from 2 to 4, and the highest Δ*K* value was observed between *K* = 2, 4, and 6 (Figure 2A). The average value of *LnP(K)* increased when the *K-value* ranged from 1 to 10 (Figure 2B), but when the *K-value* was 4, the growth rate of *LnP(K)* decreased. Therefore, the optimal *K-value* in the present study was 4 with the division of the natural population into four subgroups (Figure 2C). According to the results of the dendrogram and structure analysis, four main groups were clustered, suggesting that the four-clade model sufficiently explained the genetic structure of the 955 germplasms.

### Establishment of Molecular Identity

According to PIC value, the character of s28 was first used to distinguish 955 germplasm, followed by s25, s74 and so on. The results showed that 11 markers (s28, s25, s74, s89, s68, s30, s13, s100, s72, s77, and s3) could distinguish the 955 germplasms. The molecular identity of each germplasm consisted of 11 characters (Supplementary Table 2); for example, the molecular identity of TJ1 and TJ2 was D0002D21100 and D1002322100, respectively.

### Extraction of a Core Collection

Stepwise clustering was used to extract a core collection and using *N*_*a*_, Nei’s distance, *H*_*O*_, and PIC over the 28 SSR markers as indicators. The core collection consisted of 164 genetic individuals (Table 2), representing only 17.2% of the original genetic population. In comparison with the total natural population, the final core collection showed 94.2, 98.7, 116.4, and 116.73% of the variation based on *N*_*a*_, *H*_*O*_, Nei’s distance, and PIC, respectively. To verify the reliability of the results, three false core collections composed of 164 individuals were randomly selected, and four genetic diversity indexes (*N*_*a*_, *H*_*O*_, Nei’s distance, and PIC) of three false core collections were analyzed (Supplementary Tables 3–6). As shown in Table 3, all four indicators were significantly different from those in the truly established core collection. These results support the validity of the method for extracting the core collection and further suggest that the core collection effectively represents the entire genetic population.

**TABLE 2:** Members and molecular identity card of core collection.

**Name**	**ID**	**Name**	**ID**	**Name**	**ID**	**Name**	**ID**	**Name**	**NA**
**TJ2**	D1002322100	**TJ174**	3A00232D001	**TJ427**	D20DDBADA0B	**TJ572**	42A00123201	**TJ703**	D320AD32A0A
**TJ5**	202023AA10B	**TJ179**	4300213200A	**TJ434**	D3DD02BD20B	**TJ573**	32200A2320B	**TJ727**	3220402100A
**TJ7**	20A021A1100	**TJ181**	40004322001	**TJ446**	31020D32201	**TJ575**	DD20D3AD10A	**TJ735**	2222132D00A
**TJ9**	B0002AD2000	**TJ208**	BB00322D00A	**TJ448**	DFD20D2220A	**TJ582**	F110DA0220A	**TJ741**	AD20AA2200B
**TJ19**	DA0032D110A	**TJ209**	FF00333200A	**TJ450**	3A0203D2A0A	**TJ585**	3320DA12001	**TJ751**	2230DD2D00B
**TJ24**	B02012B100A	**TJ216**	2000232A00B	**TJ453**	D2B20D32201	**TJ594**	02002410001	**TJ779**	AA3122BD00A
**TJ27**	22202D3AA0B	**TJ217**	DA00223200B	**TJ455**	DD030B33201	**TJ597**	2120220D00B	**TJ786**	11311D2200A
**TJ28**	AD0033A200A	**TJ259**	4123D222221	**TJ478**	02D20332201	**TJ603**	220012A2200	**TJ787**	AAA12223001
**TJ32**	DD002D22A00	**TJ264**	3AA1222321B	**TJ480**	A414023020A	**TJ606**	D130AF2D00A	**TJ791**	3D1121A200A
**TJ34**	C220132B00A	**TJ265**	EA21A12211A	**TJ491**	02101223A0C	**TJ608**	0120B22DA0A	**TJ794**	D3111AAD00A
**TJ39**	D0200B32000	**TJ270**	3132A23D21B	**TJ496**	A2102A2230A	**TJ609**	A230AB2220A	**TJ802**	0BA0022220A
**TJ48**	33A00D21000	**TJ284**	3320131221A	**TJ498**	AA30222DD0B	**TJ613**	3D20032A000	**TJ806**	0A30022210A
**TJ49**	F3000221201	**TJ295**	BAA213D112A	**TJ499**	A2102322D0B	**TJ616**	3B20A2BB10A	**TJ813**	01A0A2D210A
**TJ59**	3D00232A000	**TJ296**	D3A01321211	**TJ502**	DD102222D0A	**TJ629**	421002A220A	**TJ826**	0FA0A32D10A
**TJ63**	C2202BA3100	**TJ308**	2D10DD3121A	**TJ509**	22A02B3D30B	**TJ640**	F3200421003	**TJ832**	0DA01DA020A
**TJ84**	22000A23B2A	**TJ311**	D222332101B	**TJ510**	A3202D22D0A	**TJ653**	2120DA3320A	**TJ837**	0A40B2A010A
**TJ89**	EAA04AA221A	**TJ323**	220A12BA000	**TJ512**	BA3013A3A0A	**TJ655**	AD202A1020A	**TJ852**	03D0033D10A
**TJ97**	21F00BB2031	**TJ329**	A20DAC2D00D	**TJ515**	0A20223230A	**TJ663**	03201A2320A	**TJ858**	0230032020D
**TJ102**	2D2002A220A	**TJ342**	C2AF03BF001	**TJ518**	BD102BDDA01	**TJ667**	2120222210A	**TJ861**	0130D33010A
**TJ105**	2320022212A	**TJ348**	2A430E3300A	**TJ521**	211023B2D0A	**TJ668**	121032ABA0A	**TJ867**	02002233301
**TJ109**	4130022A011	**TJ349**	2A230B33001	**TJ522**	AA302DB2D0A	**TJ669**	2B20BA30D0A	**TJ873**	0240012210B
**TJ114**	EFF0032D22A	**TJ359**	32230AA3001	**TJ524**	03202ABDA0A	**TJ670**	3330113020A	**TJ875**	0DD0032210A
**TJ116**	3030032211B	**TJ371**	322A1A2A000	**TJ529**	23A0222D201	**TJ671**	01300320D0A	**TJ882**	BAA03D3100A
**TJ124**	4130423E010	**TJ374**	024D0FDD00D	**TJ534**	0A202B3DA01	**TJ676**	A3002113A0A	**TJ890**	2A2012A200A
**TJ131**	A200A2D211A	**TJ380**	A2320F3200A	**TJ545**	332021A230A	**TJ687**	302011D020A	**TJ895**	2A202A2A00A
**TJ132**	EDA002A2230	**TJ381**	D21300B3000	**TJ546**	2A102322D0A	**TJ690**	23302422202	**TJ899**	10303D2300A
**TJ137**	DFD03DD202A	**TJ394**	B20DDFDD00A	**TJ552**	0220023DA0B	**TJ692**	B34022AD30A	**TJ930**	2C302FD200A
**TJ139**	BF4002D2231	**TJ397**	C2024EA200D	**TJ554**	011002A330B	**TJ693**	20200DA000A	**TJ932**	2A30A020001
**TJ152**	2300032211A	**TJ398**	EA322EB200A	**TJ557**	2110023230A	**TJ696**	02101D3B200	**TJ938**	2A10132000A
**TJ153**	2A2002AB02A	**TJ413**	1A2401D4102	**TJ561**	2A1001D100A	**TJ697**	2200A03220A	**TJ942**	31B0343300B
**TJ160**	33D0023202A	**TJ414**	D233022320A	**TJ562**	ED302A0A10B	**TJ698**	213023B2201	**TJ944**	2A102DB300A
**TJ166**	3200122A00A	**TJ421**	3D2D03DD301	**TJ567**	31103D02200	**TJ699**	3B3042D2A00	**TJ945**	1D10302400A
**TJ173**	2A00242200A	**TJ422**	21010D3110A	**TJ568**	FA2041A2100	**TJ701**	23302D32A00		

**TABLE 3:** Differences between genetic diversity of core collection and pseudo-core collection.

	**Na**	**Ho**	**Nei’s**	**PIC**
Core collection	3.5000	0.2351	0.5356	0.4753
First random	3.3929	0.2333	0.4436	0.3932
Second random	3.3214	0.2374	0.4672	0.4154
Third random	3.4286	0.2399	0.4524	0.4018
*P* value	<0.001	<0.001	<0.001	<0.001

## Discussion

Progress in *A. trifoliata* breeding has been slow, in part because the plants grow slowly, and new plants do not bear fruit for 4 years (Zhang et al., 2013). Therefore, cultivating new *A. trifoliata* varieties is time consuming. Furthermore, little known about the biological characteristics of *A. trifoliata*, making it difficult to select good parents for breeding. Molecular genetic markers are widely used in plant breeding, and genetic diversity must be considered when identifying trait populations and selecting parent material to ensure breeding success. The results obtained in this study can deepen our understanding of the genetic diversity of germplasm and facilitate its rational utilization of *A. trifoliata*. In this study, an SSR analysis of 955 *A. trifoliata* germplasms was performed to evaluate genetic diversity. The 28 SSR markers were selected which were high polymorphism and high stability from 49 SSR markers (Niu et al., 2019). In a previous study, 49 pairs of SSR markers were used to analyze 88 *A. trifoliata* germplasms, which were collected from eight provinces including 16 regions in China (Niu et al., 2019); PIC and H_*O*_ values were 0.43 and 0.2210, respectively, similar to those in our study (PIC = 0.41; *H*_*O*_ = 0.2382), thereby verifying that the species is moderately polymorphic (0.25 < PIC < 0.5). Additionally, 10 pairs each of ISSR and SRAP markers have been used to evaluate polymorphisms in 242 individuals from 11 natural populations (Zhang et al., 2021), resulting in genetic diversity of *N*_*a*_ = 1.99, *I*^∗^ = 0.47, ISSR; *N*_*a*_ = 1.99, and *I*^∗^ = 0.50, SRAP. These values were inferior to the corresponding values in the present study, probably because the germplasms came from only one province of China. As for genetic structure, Niu et al. (2019) divided germplasms from eight provinces into four groups, which was similar to the results of the present study. Zhang et al. (2021) divided germplasms from 11 populations into three groups. These studies indicated that the genetic diversity of *A. trifoliata* was weak.

Although crop breeding is based on abundant crop germplasm resources, redundant germplasms have various limitations. For example, it is difficult to precisely and rapidly identify useful resources for plant breeding. The management and preservation of germplasm resources are expensive and time-consuming, a core collection can effectively resolve these issues (Frankel and Brown, 1984). Although core collections have been reported for *A. trifoliata* (56/242), the germplasm was only from the Qinba mountain area of China, and the results had certain limitations (Zhang et al., 2021). The core germplasm represented 17.1% of all accessions, which is higher than the range of 5–10% recommended by Brown as well as the values reported in other plants (Brown, 1989), e.g., *sesame (S. indicum)* (28/277) (Park et al., 2014), *maize (Z. mays)* (951/13521) (Yu et al., 2005), and *soyabean (G.* max) (Oliveira et al., 2010). In contrast, they are slightly less than those for the *rubber tree (Hevea brasiliensis)* (128/505) (Adifaiz et al., 2020), *ramie (Boehmeria nivea L.)* (22/105) (Luan et al., 2014), and *Gympie messmate (Eucalyptus cloeziana F. Muell., family Myrtaceae Juss.)* (247/707) (Lv et al., 2020). However, in applying one additional filter, the N_*a*_ and H_*O*_ are reduced to 82.1 and 84.2%, respectively, of those for the full population, and the core collection is reduced to eight genetic individuals. The maintenance of the vast majority of germplasm diversity should be a priority for guiding the determination of an optimal fraction; accordingly, we did not aim for a low rate of germplasm retention. In comparison with the total natural population, the final core collection retention rate was 94.2, 98.7, 116.4, and 116.73% of Na, H_*O*_, Nei’s distance, and PIC, respectively. These results indicated that the core collection could represent the genetic diversity of 955 germplasms.

Abundant crop germplasm resources are the basis of crop breeding, but germplasm collections is time demanding and laborious. Collection of the 955 germplasm resources from China took about 10 years, and the germplasms were cultivated in the Taojiang experimental field of the Institute of Bast Fiber Crops, Chinese Academy of Agricultural Sciences. However, due to early management and staff turnover, the geographic origin of each germplasm was unclear, which limits our understanding and utilization of the germplasm resources. Although the geographic origin of germplasms is important for genetic diversity research, the 955 germplasms in our collection are still important for the breeding of *A. trifoliata*, even with some missing geographic information. The use of modern technology to distinguish these germplasms is a key possibility. SSR molecular marker technology is not affected by geographical origin and can result in high polymorphism, stable results, and good repeatability (Ni et al., 2018). Therefore, SSR markers were used in the present study. Based on our results, we could reclassify these germplasms into individuals and populations. Thus, the molecular identity and core collection will be useful for management strategies, and the genetic diversity and genetic structure will be beneficial for breeding.

## Conclusion

To the best of our knowledge, this study used the largest number of *A. trifoliata* germplasms to date. This results showed moderate genetic diversity and weak genetic structure in the natural population of *A. trifoliata*, based on 28 SSR markers. Eleven pairs of primers could distinguish the 955 germplasms, and the molecular identity of each germplasm consisted of 11 characters. A core germplasm collection consisting of 164 germplasms was generated, accounting for 17.2% of the original germplasm. Further, estimated of genetic diversity and genetic structure could provide a foundation for future *A. trifoliata* breeding. The core collection and molecular identity could reduce the management cost and improve the protection of germplasm resources.

## Data Availability Statement

The raw data supporting the conclusions of this article will be made available by the authors, without undue reservation.

## Author Contributions

ML and JaC conceived and designed the project. YZ, YW, ZS, JN, YS, KH, and JnC collected the plant materials. YZ, YW, ZS, JN, YS, and JnC performed molecular labwork and scored the markers. YZ and YW analyzed the data and wrote the manuscript with assistance from all other authors. All authors read and approved the final manuscript.

## Conflict of Interest

The authors declare that the research was conducted in the absence of any commercial or financial relationships that could be construed as a potential conflict of interest.

## Publisher’s Note

All claims expressed in this article are solely those of the authors and do not necessarily represent those of their affiliated organizations, or those of the publisher, the editors and the reviewers. Any product that may be evaluated in this article, or claim that may be made by its manufacturer, is not guaranteed or endorsed by the publisher.

## Supplementary Material

Supplementary Table 1 Characteristics of 28 SSR primers.

Supplementary Table 2 Molecular identity of the 955 *A. trifoliate* accessions.

Supplementary Table 3 N_*a*_ in core collection and random core collection at 28 SSR markers.

Supplementary Table 4 Ho in core collection and random core collection at 28 SSR markers.

Supplementary Table 5 Nei’s in core collection and random core collection at 28 SSR markers.

Supplementary Table 6 PIC in core collection and random core collection at 28 SSR markers.

## Abbreviations

SSR: simple sequence repeat
N_*a*_: number of alleles
N_*e*_: effective number of alleles
H_*o*_: observed heterozygosity
H_*e*_: expected heterozygosity
Nei’ s: genetic distance
I^∗^: Shannon’s information index
PIC: polymorphic information content.
