# Supplementary material for: Genetic Diversity of a Natural Population of Akebia trifoliata (Thunb.) Koidz and Extraction of a Core Collection Using Simple Sequence Repeat Markers
Source: Front Genet. 2021 Aug 31;12:716498. doi: 10.3389/fgene.2021.716498 (PMC8438410; doi:10.3389/fgene.2021.716498)
Supplement: Supplementary Table 1 — Characteristics of 28 SSR primers. [file Table_1.doc]

**Supplementary Table S1**

**Characteristics of 28 SSR primers.**

| Marker | Gene_ID | Repeat motif | Primer pair (5’-3’) | Size (bp) |
| --- | --- | --- | --- | --- |
| s3 | c14599_c1_g3 | (GAC)5 | AACGCCTCCAAAGGGATGAG | 252 |
|  |  |  | GAGGAGATGAGGTGAAGCCG |  |
| s4 | c6766_c0_g1 | (TTC)5 | GCGCTCTTCTCTTCTGTGGT | 231 |
|  |  |  | GGTAGTTCGGTGCCAGTTGA |  |
| s5 | c11874_c0_g1 | (AG)7 (G)11 | CAACCAATTGCTGCCCATGG | 177 |
|  |  |  | GCGCTCTATCTTGGCTGGAT |  |
| s13 | c1927_c0_g1 | (TC)11 | ATGGGTTGTGAAGCGCCATA | 215 |
|  |  |  | ACACCAGCCTGTTCTAGTGC |  |
| s19 | c37961_c0_g1 | (TG)6(TC)6 | AGTGCCTAACCAACCCATGT | 191 |
|  |  |  | GCGCAGAGCTTCGATTCTTG |  |
| s22 | c14528_c1_g1 | (CCA)5 | AGCTGGGCTAAGTTGGCAAA | 216 |
|  |  |  | GTGTCATCGCTCTCGCTGTA |  |
| s24 | c5508_c0_g1 | (CT)8 (AG)7 | GGCTGAGCCCTCATACACTT | 280 |
|  |  |  | GCTTTGGGCATAAACGACGA |  |
| s25 | c6825_c0_g1 | (TC)19 | GAGGCCAGCTTTACAGAGCA | 242 |
|  |  |  | GTCAAGAGAGAGGTGGTGCA |  |
| s27 | c13580_c0_g1 | (CAT)8 | GCCCAAGTGTCCAATTGTCC | 230 |
|  |  |  | CGTACCCAACCACGTACCAA |  |
| s28 | c13090_c1_g2 | (TC)9 | GAGGTGGAGATGGAGGAGGA | 216 |
|  |  |  | AACCCTAGTTCTCTCTGCGT |  |
| s30 | c14482_c0_g3 | (AG)16 | AGCAACCGAAGATTCTGCCA | 158 |
|  |  |  | CTTCAGACGCTCACACTCGT |  |
| s32 | c6144_c0_g1 | (AG)11 | TGTGCGAAGGAGGAAGTGTC | 163 |
|  |  |  | CTGCCCAAGTACTAACCGGT |  |
| s34 | c13887_c6_g2 | (TTTTG)5 | TGGAGATGTTGAAATCCCACA | 278 |
|  |  |  | GCTCCGAACTGACTAGATCCA |  |
| s40 | c13725_c4_g1 | (CCA)7 | AAATACACTGTCGACTTCCGA | 217 |
|  |  |  | TCCCTCTCTGCTTCGAGTGA |  |
| s46 | c1000_c0_g1 | (CT)9 | TACAAGTGGGCGGAGATGAC | 191 |
|  |  |  | ACACATGCTAAAAGGCCGGT |  |
| s50 | c14479_c0_g2 | (TCA)5 | GGACTCGTTGGAACAGACGT | 266 |
|  |  |  | TCTCAACACCTTCCTCCCCA |  |
| s52 | c13640_c0_g1 | (GA)10 | CACCAATCACAGGCGTTGC | 141 |
|  |  |  | AACCCGGGTACATGTTTGCA |  |
| s57 | c14561_c4_g3 | (TC)8 | CTCTCTCGTCTTTGCTGCCA | 181 |
|  |  |  | CGGTTTGGTCGTAAATCGCA |  |
| s59 | 2584_c0_g1 | (CT)11 | AGGTGTAGACGAGGTCCTCC | 198 |
|  |  |  | TCGCCATCCAAATCTACCATT |  |
| s67 | c6691_c0_g1 | (GAA)8 | GAGATCCACAAGCGAGCGAA | 196 |
|  |  |  | AGCCGCGTTATTCAATCCCT |  |
| s68 | c10956_c0_g1 | (TC)13 (TA)6 | GCATGTAACACCCCATCCCA | 254 |
|  |  |  | ACGTGCCATTCCAAGGAGAA |  |
| s72 | c13823_c4_g3 | (TGC)7 | AGCTTTGATGGGGATGGGTG | 250 |
|  |  |  | CTCTCAGGTTAGCCCGCATT |  |
| s74 | c13519_c0_g1 | (TC)14 | GGCGACTTACCTGATCCGAG | 160 |
|  |  |  | GCGATCATCTTTCGGCAACC |  |
| s77 | c15128_c1_g1 | (AGG)5 | CAGCAACACCAAGAAGCCAC | 240 |
|  |  |  | ATCTAAGGGCGGACCAGAGT |  |
| s78 | c15128_c1_g1 | (TTA)5 | GCGACCATGAAACCCTAGCT | 267 |
|  |  |  | CCCTCACATCTCAACTAGCGG |  |
| s84 | c14885_c1_g4 | (AG)9 | ACAGGTACATCAACGCGGTT | 203 |
|  |  |  | GCAAACCCAATCCCCATCCT |  |
| s89 | c15100_c2_g1 | (AG)10 | GGTTTGGTGCCTGATGACCT | 185 |
|  |  |  | CTTCAGCCCAGTCGTGACTT |  |
| s90 | c12812_c2_g2 | (CCA)5 | TGGTTCATCTTGGAGTGCGT | 208 |
|  |  |  | TCTGGAAGGAAGGGAGGGAG |  |
| s91 | c12812_c2_g2 | (TGC)5 | TGGTTCATCTTGGAGTGCGT | 194 |
|  |  |  | AGACTTCTCAGGCACACGTG |  |
| s92 | c12812_c2_g2 | (AAGA)5 | CACGTGTGCCTGAGAAGTCT | 250 |
|  |  |  | CACAGCCGATCCTTCCAACT |  |
| s96 | c15106_c0_g3 | (CAT)6 | GCGGTACATTCCCCGGTAAA | 241 |
|  |  |  | ACTCGTAATCCTGTTGGCCG |  |
| s100 | c13025_c2_g1 | (CAA)6 | GTTCGCGTGGGAAGAACAAC | 217 |
|  |  |  | ACCTTCTGATGCGCTTCGAA |  |
