# Supplementary material for: Genetic Diversity of a Natural Population of Akebia trifoliata (Thunb.) Koidz and Extraction of a Core Collection Using Simple Sequence Repeat Markers
Source: Front Genet. 2021 Aug 31;12:716498. doi: 10.3389/fgene.2021.716498 (PMC8438410; doi:10.3389/fgene.2021.716498)
Supplement: Supplementary Table 2 — Molecular identity of the 955 A. trifoliate accessions. [file Table_2.doc]

S**upplementary Table 2**

**Molecular identity card of 955 A.trifoliata germplasms**

| **Name** | **ID** | **Name** | **ID** | **Name** | **ID** | **Name** | **ID** | **Name** | **ID** |
| --- | --- | --- | --- | --- | --- | --- | --- | --- | --- |
| **TJ1** | D0002D21100 | **TJ38** | A0A00021100 | **TJ75** | DD002DDD00A | **TJ112** | DB0001A001A | **TJ149** | 4BD0A32211B |
| **TJ2** | D1002322100 | **TJ39** | D0200B32000 | **TJ76** | 4F302D2B10A | **TJ113** | E3D0032A12A | **TJ150** | DAA0A22213A |
| **TJ3** | 33003D11200 | **TJ40** | D01002B1000 | **TJ77** | B2A022A220A | **TJ114** | EFF0032D22A | **TJ151** | DA400D22021 |
| **TJ4** | 30002DD310B | **TJ41** | D200032110A | **TJ78** | 3D201DA210A | **TJ115** | 3030A22A21A | **TJ152** | 2300032211A |
| **TJ5** | 202023AA10B | **TJ42** | B22002DA00D | **TJ79** | E3002312100 | **TJ116** | 3030032211B | **TJ153** | 2A2002AB02A |
| **TJ6** | 102023AA10B | **TJ43** | CDA00AB2A0A | **TJ80** | 0300032220D | **TJ117** | A1D0D3A211A | **TJ154** | 3FA0A232021 |
| **TJ7** | 20A021A1100 | **TJ44** | DAA00AD200A | **TJ81** | A300032BA0A | **TJ118** | A110021DA3A | **TJ155** | 2A40D222A2B |
| **TJ8** | A00012B2A00 | **TJ45** | AD200D3A00A | **TJ82** | 2D203222D2A | **TJ119** | 433002A222A | **TJ156** | DA00DDA320A |
| **TJ9** | B0002AD2000 | **TJ46** | AD20023A00A | **TJ83** | 22000320D2A | **TJ120** | DD300D321AB | **TJ157** | 23A0122DA3A |
| **TJ10** | E00032A2100 | **TJ47** | 23A0030A000 | **TJ84** | 22000A23B2A | **TJ121** | 3D0002ABA21 | **TJ158** | 23A0122DA2A |
| **TJ11** | 200032A1000 | **TJ48** | 33A00D21000 | **TJ85** | F30003A2A1A | **TJ122** | 4D00023212B | **TJ159** | E4A0022301A |
| **TJ12** | A01012D1001 | **TJ49** | F3000221201 | **TJ86** | DBA0A22BA3A | **TJ123** | B300A2A2211 | **TJ160** | 33D0023202A |
| **TJ13** | D0102A22001 | **TJ50** | 2D0002AA001 | **TJ87** | DD303BAD23A | **TJ124** | 4130423E010 | **TJ161** | D0002A2A00A |
| **TJ14** | 10002DBB201 | **TJ51** | 2B002AA120B | **TJ88** | FB003D2210A | **TJ125** | D200A22202A | **TJ162** | AE00132D00B |
| **TJ15** | F0003321200 | **TJ52** | 2A202D2200B | **TJ89** | EAA04AA221A | **TJ126** | DD00032201A | **TJ163** | DA00AA2A001 |
| **TJ16** | 4300DD21100 | **TJ53** | 3D20323A20A | **TJ90** | DA2002A213A | **TJ127** | D30002AD3AA | **TJ164** | A100AA2D00A |
| **TJ17** | 2000AADA000 | **TJ54** | 2F2022B110A | **TJ91** | DD0002D012A | **TJ128** | 32400D23211 | **TJ165** | D000131E00A |
| **TJ18** | AA001DB1A02 | **TJ55** | F220222A200 | **TJ92** | CB20A3B211A | **TJ129** | DD000222121 | **TJ166** | 3200122A00A |
| **TJ19** | DA0032D110A | **TJ56** | 31100D2D000 | **TJ93** | B33002222DA | **TJ130** | 3200022B231 | **TJ167** | 2200313200A |
| **TJ20** | AD0022BA20A | **TJ57** | 2D00DA1D00B | **TJ94** | B4100232A2A | **TJ131** | A200A2D211A | **TJ168** | 3200233D001 |
| **TJ21** | AA000D2A00A | **TJ58** | FD00022D00B | **TJ95** | 2D400323131 | **TJ132** | EDA002A2230 | **TJ169** | 2200AB2200A |
| **TJ22** | D3A0222A20A | **TJ59** | 3D00232A000 | **TJ96** | BF0002A2111 | **TJ133** | DF30D322221 | **TJ170** | DA00ADA200A |
| **TJ23** | B0A0A32110A | **TJ60** | 0D00D0D000B | **TJ97** | 21F00BB2031 | **TJ134** | EA00022D221 | **TJ171** | B00032AD00A |
| **TJ24** | B02012B100A | **TJ61** | 000020DA00B | **TJ98** | E2A0022AD2A | **TJ135** | 2B302D22200 | **TJ172** | E000D2AD00A |
| **TJ25** | 3020DDAA200 | **TJ62** | D02032A1101 | **TJ99** | 2CC00232021 | **TJ136** | 2D400332001 | **TJ173** | 2A00242200A |
| **TJ26** | DA2032AA10A | **TJ63** | C2202BA3100 | **TJ100** | 432002222AA | **TJ137** | DFD03DD202A | **TJ174** | 3A00232D001 |
| **TJ27** | 22202D3AA0B | **TJ64** | DA003DA1200 | **TJ101** | B1A0032001A | **TJ138** | 4D20032212A | **TJ175** | 2D00232D00A |
| **TJ28** | AD0033A200A | **TJ65** | E2002D11100 | **TJ102** | 2D2002A220A | **TJ139** | BF4002D2231 | **TJ176** | 3D00342D00A |
| **TJ29** | F4002DA200B | **TJ66** | 040012D220A | **TJ103** | BB20022212A | **TJ140** | DA300202A30 | **TJ177** | B300232200A |
| **TJ30** | DE00223300A | **TJ67** | A300A32A00B | **TJ104** | 2300022D001 | **TJ141** | DA40D223121 | **TJ178** | A100222A00A |
| **TJ31** | EA003D1210A | **TJ68** | E31010B110A | **TJ105** | 2320022212A | **TJ142** | ADB0A22222A | **TJ179** | 4300213200A |
| **TJ32** | DD002D22A00 | **TJ69** | DC201122A0A | **TJ106** | 3D1002AD02A | **TJ143** | EB200122111 | **TJ180** | BB0012AA00A |
| **TJ33** | DA003B22000 | **TJ70** | 3D203BB1A00 | **TJ107** | DBB00223A21 | **TJ144** | 3B0002321AB | **TJ181** | 40004322001 |
| **TJ34** | C220132B00A | **TJ71** | 30001D20200 | **TJ108** | C1D00A2212A | **TJ145** | 4D300332211 | **TJ182** | DA00ABA300A |
| **TJ35** | 3120022A00B | **TJ72** | A1001D12300 | **TJ109** | 4130022A011 | **TJ146** | 2A400232021 | **TJ183** | 4B00322300A |
| **TJ36** | 210002A110A | **TJ73** | 3B00A232100 | **TJ110** | 2A00031DA0A | **TJ147** | 3D30E222121 | **TJ184** | DC00322D001 |
| **TJ37** | 0D00022100A | **TJ74** | 330023B2200 | **TJ111** | B3A0032D0AD | **TJ148** | 4B303232220 | **TJ185** | B2002D3D00A |

| **TJ166** | 3200122A00A | **TJ205** | 3A0033A200A | **TJ244** | 2A30022321A | **TJ283** | 3A22332D22A | **TJ322** | D20D2D3D00A |
| --- | --- | --- | --- | --- | --- | --- | --- | --- | --- |
| **TJ167** | 2200313200A | **TJ206** | 040032AD00A | **TJ245** | D2A22122A1A | **TJ284** | 3320131221A | **TJ323** | 220A12BA000 |
| **TJ168** | 3200233D001 | **TJ207** | 3B0032D200A | **TJ246** | 2303222011D | **TJ285** | DA1A121221A | **TJ324** | B20D0B2D000 |
| **TJ169** | 2200AB2200A | **TJ208** | BB00322D00A | **TJ247** | 3301012002A | **TJ286** | 3D10222221A | **TJ325** | BD2DD03D000 |
| **TJ170** | DA00ADA200A | **TJ209** | FF00333200A | **TJ248** | 3AAA0222011 | **TJ287** | 3122232D21A | **TJ326** | D2DD2DAD00A |
| **TJ171** | B00032AD00A | **TJ210** | 3B00223A00A | **TJ249** | 2D3202A2021 | **TJ288** | D12313A221A | **TJ327** | B20D2B3D00A |
| **TJ172** | E000D2AD00A | **TJ211** | 3A0032B200B | **TJ250** | DAA20A2001B | **TJ289** | 2A11232021A | **TJ328** | B2020022001 |
| **TJ173** | 2A00242200A | **TJ212** | AE0022D200A | **TJ251** | 2D13322012A | **TJ290** | DDA0A30011A | **TJ329** | A20DAC2D00D |
| **TJ174** | 3A00232D001 | **TJ213** | 3A002B2D00B | **TJ252** | 4A012122A1A | **TJ291** | 03A2B3A0111 | **TJ330** | 2D3A02DA00B |
| **TJ175** | 2D00232D00A | **TJ214** | 4A00A2D2001 | **TJ253** | 4D01312221A | **TJ292** | D120D30121A | **TJ331** | 320D042D000 |
| **TJ176** | 3D00342D00A | **TJ215** | 33001332001 | **TJ254** | 3202322222A | **TJ293** | ADD3132122A | **TJ332** | 322A0D2A000 |
| **TJ177** | B300232200A | **TJ216** | 2000232A00B | **TJ255** | DBA1A2A021A | **TJ294** | EAA2A33122A | **TJ333** | E222AA2200A |
| **TJ178** | A100222A00A | **TJ217** | DA00223200B | **TJ256** | 3203022001A | **TJ295** | BAA213D112A | **TJ334** | 323D0D2D00A |
| **TJ179** | 4300213200A | **TJ218** | F30024A000A | **TJ257** | ABB23A20A2A | **TJ296** | D3A01321211 | **TJ335** | 222D0E2D00A |
| **TJ180** | BB0012AA00A | **TJ219** | 3A00D2A000A | **TJ258** | 3A01022A11A | **TJ297** | DA32132122A | **TJ336** | 2A420E22000 |
| **TJ181** | 40004322001 | **TJ220** | EF00022000A | **TJ259** | 4123D222221 | **TJ298** | BAD2121112A | **TJ337** | E20DA32D00A |
| **TJ182** | DA00ABA300A | **TJ221** | BA00A3BD001 | **TJ260** | 0D030220021 | **TJ299** | AAD0D22122A | **TJ338** | DA1B02AB00A |
| **TJ183** | 4B00322300A | **TJ222** | ED003223001 | **TJ261** | 3A02A21012D | **TJ300** | 21202D2120B | **TJ339** | D2B21D2200A |
| **TJ184** | DC00322D001 | **TJ223** | D400222D00D | **TJ262** | 4A21022222A | **TJ301** | C2202A1220A | **TJ340** | B2BA0DBA00A |
| **TJ185** | B2002D3D00A | **TJ224** | 3A00130A00A | **TJ263** | B21123A011A | **TJ302** | 2D31DD3121A | **TJ341** | D2AD2D2D00A |
| **TJ186** | DA0033A200A | **TJ225** | 2A0032A200A | **TJ264** | 3AA1222321B | **TJ303** | 2222AD1121A | **TJ342** | C2AF03BF001 |
| **TJ187** | A000230A00A | **TJ226** | 2B00A23B00A | **TJ265** | EA21A12211A | **TJ304** | DA23122122A | **TJ343** | BA224DD200A |
| **TJ188** | E300D30E00A | **TJ227** | DC003D1200A | **TJ266** | D2D1DD1211A | **TJ305** | DA220A2112A | **TJ344** | 3232233200B |
| **TJ189** | B2001AA200A | **TJ228** | D3000D12001 | **TJ267** | F2212DA321A | **TJ306** | E2100AA122A | **TJ345** | D2223AD200A |
| **TJ190** | 3A003F22001 | **TJ229** | DA00DF22001 | **TJ268** | 22B0222021A | **TJ307** | 2AB2DA0121A | **TJ346** | EAD22C3200A |
| **TJ191** | 2B00222200A | **TJ230** | DF003D2D001 | **TJ269** | 2211D2A021A | **TJ308** | 2D10DD3121A | **TJ347** | 223D20DD001 |
| **TJ192** | BD0012D200A | **TJ231** | 23003A22001 | **TJ270** | 3132A23D21B | **TJ309** | 2102A321A1A | **TJ348** | 2A430E3300A |
| **TJ193** | 3B00A22200A | **TJ232** | EB001A2200A | **TJ271** | 2A31322D12B | **TJ310** | 312013A1A1A | **TJ349** | 2A230B33001 |
| **TJ194** | BA0002AD00A | **TJ233** | 2C00A32300A | **TJ272** | 4123322B121 | **TJ311** | D222332101B | **TJ350** | DA423EB2001 |
| **TJ195** | 2300232200A | **TJ234** | 3A001D2200B | **TJ273** | 3A21020D22B | **TJ312** | BA20333121A | **TJ351** | DA0A033A00A |
| **TJ196** | B300A21200A | **TJ235** | 31003222000 | **TJ274** | 32A2122D12A | **TJ313** | 2A313A2A22A | **TJ352** | 22023FD200A |
| **TJ197** | 4D00D12200A | **TJ236** | DD00AD2200A | **TJ275** | CDA2112A22A | **TJ314** | D203A22221A | **TJ353** | 3A02A22200A |
| **TJ198** | 30002D2200A | **TJ237** | EB00212B00D | **TJ276** | 2320A32221A | **TJ315** | 2312D2AD11A | **TJ354** | 320E032E00A |
| **TJ199** | 3000212300A | **TJ238** | DF00232200A | **TJ277** | 3AD02322221 | **TJ316** | B220222221A | **TJ355** | 323203D200A |
| **TJ200** | 3000322200A | **TJ239** | 2A00DD2200A | **TJ278** | 3320222D22A | **TJ317** | D123122D11A | **TJ356** | 220232D2000 |
| **TJ201** | BD0002A200D | **TJ240** | DA003D22001 | **TJ279** | E2A3202A12D | **TJ318** | D1021DAD21B | **TJ357** | 2A0A0B2A00A |
| **TJ202** | EA00D3AA00D | **TJ241** | 22B0302022A | **TJ280** | AAA22D21A1A | **TJ319** | ED33A23201A | **TJ358** | 320B033B00A |
| **TJ203** | D3000DA200A | **TJ242** | A222012002B | **TJ281** | 2D132D2B11A | **TJ320** | DA200332A1A | **TJ359** | 32230AA3001 |
| **TJ204** | D000A32200B | **TJ243** | 2A120220011 | **TJ282** | 2BA3122021A | **TJ321** | 20020032000 | **TJ360** | A20D0D3D00A |

| **TJ361** | D20AAEDA001 | **TJ400** | 0102DA0200D | **TJ439** | 2A2D023D10A | **TJ478** | 02D20332201 | **TJ517** | 03102322D0A |
| --- | --- | --- | --- | --- | --- | --- | --- | --- | --- |
| **TJ362** | B20A0DDA00A | **TJ401** | DA02020220A | **TJ440** | A2AD0DAD10A | **TJ479** | AD33023310A | **TJ518** | BD102BDDA01 |
| **TJ363** | 32A20DD2000 | **TJ402** | 3DB222A220A | **TJ441** | 03030AD310A | **TJ480** | A414023020A | **TJ519** | 22302D22A0D |
| **TJ364** | A2020FB2001 | **TJ403** | 310D1AAD101 | **TJ442** | 033A0DBA201 | **TJ481** | 2A100A2A30A | **TJ520** | 22303DBAD01 |
| **TJ365** | B2023DD2000 | **TJ404** | 320D22AD20A | **TJ443** | DDD2023220A | **TJ482** | 21202220D0B | **TJ521** | 211023B2D0A |
| **TJ366** | D2430F33001 | **TJ405** | 2A022A0220A | **TJ444** | 2A2D022D101 | **TJ483** | 2A204D30A0A | **TJ522** | AA302DB2D0A |
| **TJ367** | A2220DD200A | **TJ406** | DA12033210A | **TJ445** | 2100033020A | **TJ484** | 22100AA2D0A | **TJ523** | D2202BA2D0A |
| **TJ368** | AA33DB13001 | **TJ407** | A23B123B10A | **TJ446** | 31020D32201 | **TJ485** | 221002AD30A | **TJ524** | 03202ABDA0A |
| **TJ369** | DAA20D3200A | **TJ408** | 2D020A3210A | **TJ447** | DA030A2320A | **TJ486** | A220AD2230A | **TJ525** | 02202A32D0A |
| **TJ370** | 2A0B0A2B001 | **TJ409** | 3AA20DB210A | **TJ448** | DFD20D2220A | **TJ487** | A2100322A01 | **TJ526** | 2A102B2D30A |
| **TJ371** | 322A1A2A000 | **TJ410** | 2D2D0D2D10A | **TJ449** | A222DA3220B | **TJ488** | 2D10032A00A | **TJ527** | 02002DB200A |
| **TJ372** | D2A30A3300A | **TJ411** | D2B2020220A | **TJ450** | 3A0203D2A0A | **TJ489** | 2210132AD0A | **TJ528** | 0220A233D0A |
| **TJ373** | 22233DA3001 | **TJ412** | AAD2AD3220A | **TJ451** | 0A020B3220B | **TJ490** | AA20212DD0A | **TJ529** | 23A0222D201 |
| **TJ374** | 024D0FDD00D | **TJ413** | 1A2401D4102 | **TJ452** | AD0D0BBD20A | **TJ491** | 02101223A0C | **TJ530** | 1320223D001 |
| **TJ375** | D2120A2200A | **TJ414** | D233022320A | **TJ453** | D2B20D32201 | **TJ492** | 0210213BA01 | **TJ531** | 0230222D201 |
| **TJ376** | DA02032200A | **TJ415** | 2222022220A | **TJ454** | A3220232A0A | **TJ493** | A200202230A | **TJ532** | 0D2023DD00A |
| **TJ377** | DA0203D200A | **TJ416** | D3A20A3220A | **TJ455** | DD030B33201 | **TJ494** | 01202A22A0A | **TJ533** | 0210213200B |
| **TJ378** | D2A20FB200A | **TJ417** | DA020BA220A | **TJ456** | 2CD20B3220A | **TJ495** | 22102A2330A | **TJ534** | 0A202B3DA01 |
| **TJ379** | E2220D3200A | **TJ418** | 22AEAA2E20A | **TJ457** | 2C320D3220A | **TJ496** | A2102A2230A | **TJ535** | 0220222DA0A |
| **TJ380** | A2320F3200A | **TJ419** | 22A2DBA230A | **TJ458** | 0BC202D220A | **TJ497** | 2D1022A2D0A | **TJ536** | 0D10223BA0A |
| **TJ381** | D21300B3000 | **TJ420** | AD1D2ABD20A | **TJ459** | 2B230AB310A | **TJ498** | AA30222DD0B | **TJ537** | 3D1023DA30A |
| **TJ382** | BA42DD3200A | **TJ421** | 3D2D03DD301 | **TJ460** | 21320232A0B | **TJ499** | A2102322D0B | **TJ538** | 221023BDD0A |
| **TJ383** | DDA2E3D200A | **TJ422** | 21010D3110A | **TJ461** | D3020D2220A | **TJ500** | 02A022BD30A | **TJ539** | 01102322D0A |
| **TJ384** | D20D033D00A | **TJ423** | A2A1023110A | **TJ462** | D32D0DDD20B | **TJ501** | 0230222DD0A | **TJ540** | 02102232A0B |
| **TJ385** | BA02ABD2000 | **TJ424** | AA3B023B20A | **TJ463** | 23D20A2220A | **TJ502** | DD102222D0A | **TJ541** | 0A10A2A230A |
| **TJ386** | 222D04DD00A | **TJ425** | 32020DD220A | **TJ464** | A3420DA2101 | **TJ503** | A230A32220A | **TJ542** | 23002323A0A |
| **TJ387** | 222202A2000 | **TJ426** | 3AB202B220B | **TJ465** | A4220D3200A | **TJ504** | 22A023A2D0A | **TJ543** | 02302D02D0B |
| **TJ388** | EAB23D3200A | **TJ427** | D20DDBADA0B | **TJ466** | 23D20032001 | **TJ505** | A2102210D0A | **TJ544** | 0B20222230A |
| **TJ389** | 222233D200A | **TJ428** | 2A2D0B3D10A | **TJ467** | 00020A22001 | **TJ506** | AAA022AD30A | **TJ545** | 332021A230A |
| **TJ390** | A20B2B3B00A | **TJ429** | DD220132A0B | **TJ468** | 030D0DBD00B | **TJ507** | A220ADAD30B | **TJ546** | 2A102322D0A |
| **TJ391** | 22ADDDDD00D | **TJ430** | D2D20332A0B | **TJ469** | A402022200A | **TJ508** | A3202A2A30D | **TJ547** | 0A20230020A |
| **TJ392** | C20D0EBD001 | **TJ431** | 22210D31A0A | **TJ470** | D3020A2200A | **TJ509** | 22A02B3D30B | **TJ548** | 02202302D0A |
| **TJ393** | D2A22A2200A | **TJ432** | 230102D120A | **TJ471** | 22020DA200A | **TJ510** | A3202D22D0A | **TJ549** | 0230222A00A |
| **TJ394** | B20DDFDD00A | **TJ433** | 2201DD2120A | **TJ472** | 22A3023300A | **TJ511** | 22F0A0BA30A | **TJ550** | 0220233DD0A |
| **TJ395** | 220223A200A | **TJ434** | D3DD02BD20B | **TJ473** | DA3B022B20A | **TJ512** | BA3013A3A0A | **TJ551** | 0220032E00A |
| **TJ396** | 42D2FD2200A | **TJ435** | 222D02DDA01 | **TJ474** | 2D320DD210A | **TJ513** | 2A20233230A | **TJ552** | 0220023DA0B |
| **TJ397** | C2024EA200D | **TJ436** | D2220D4220A | **TJ475** | ADA20A3220A | **TJ514** | 02202DA230A | **TJ553** | 0120032AD0A |
| **TJ398** | EA322EB200A | **TJ437** | 22B20AD220A | **TJ476** | 2AAD020D10A | **TJ515** | 0A20223230A | **TJ554** | 011002A330B |
| **TJ399** | AAD2DD2200A | **TJ438** | DA0D0DDD20A | **TJ477** | 2EA302D320A | **TJ516** | 02102DDAD0A | **TJ555** | 01200D2D30A |

| **TJ556** | 311002AD30A | **TJ595** | 22202422000 | **TJ634** | B1200D02202 | **TJ673** | AD00202210A | **TJ712** | 0010232200A |
| --- | --- | --- | --- | --- | --- | --- | --- | --- | --- |
| **TJ557** | 2110023230A | **TJ596** | 22202422001 | **TJ635** | 0A100F22A0A | **TJ674** | 1D30A232A01 | **TJ713** | 2020043D00A |
| **TJ558** | 0120020D301 | **TJ597** | 2120220D00B | **TJ636** | 32000DAA00A | **TJ675** | BD202D23001 | **TJ714** | 30002DA200A |
| **TJ559** | 2A200D2200A | **TJ598** | 2120022D001 | **TJ637** | B1000A3A00B | **TJ676** | A3002113A0A | **TJ715** | 4D001A2200A |
| **TJ560** | 23200DA2301 | **TJ599** | 2220D32D000 | **TJ638** | 02A00D2A002 | **TJ677** | A32023D010A | **TJ716** | DF201D3200A |
| **TJ561** | 2A1001D100A | **TJ600** | 2320D22D000 | **TJ639** | 42200222002 | **TJ678** | AD20A23310B | **TJ717** | 0D303AA2001 |
| **TJ562** | ED302A0A10B | **TJ601** | 02001A2220A | **TJ640** | F3200421003 | **TJ679** | AB00AA2D20D | **TJ718** | DA402D3200A |
| **TJ563** | A220A20B10A | **TJ602** | 22000E2220A | **TJ641** | 04002000200 | **TJ680** | 0000232020B | **TJ719** | 2E101F2000A |
| **TJ564** | D220A221A00 | **TJ603** | 220012A2200 | **TJ642** | 0F30D0B220A | **TJ681** | DD00242D10A | **TJ720** | 0210AAD0000 |
| **TJ565** | 0D101A2B100 | **TJ604** | D1000D2200A | **TJ643** | 232012A0200 | **TJ682** | D3202122D0A | **TJ721** | 0130220A001 |
| **TJ566** | 22300D0AA0A | **TJ605** | 3200AD2D00A | **TJ644** | 23302AA2A0A | **TJ683** | 2B203A2DD0A | **TJ722** | 0210212A00A |
| **TJ567** | 31103D02200 | **TJ606** | D130AF2D00A | **TJ645** | 000033B1200 | **TJ684** | 21301A3AA0A | **TJ723** | D3201A2A00A |
| **TJ568** | FA2041A2100 | **TJ607** | D3200A2D00A | **TJ646** | 0A20110020A | **TJ685** | 23303A3220A | **TJ724** | A210B21B00A |
| **TJ569** | F2302D01A0B | **TJ608** | 0120B22DA0A | **TJ647** | 0B000A00200 | **TJ686** | 2B202DAAD0A | **TJ725** | 322022AA00A |
| **TJ570** | D220032120A | **TJ609** | A230AB2220A | **TJ648** | 00201D00200 | **TJ687** | 302011D020A | **TJ726** | AA102AAB00A |
| **TJ571** | A1A00131A01 | **TJ610** | D220DD1020A | **TJ649** | 01002D30001 | **TJ688** | 0B30AD30D01 | **TJ727** | 3220402100A |
| **TJ572** | 42A00123201 | **TJ611** | D120A2A220A | **TJ650** | A1D03222200 | **TJ689** | AF303D3A10A | **TJ728** | 2120303100A |
| **TJ573** | 32200A2320B | **TJ612** | 0A20133200A | **TJ651** | 01202232A00 | **TJ690** | 23302422202 | **TJ729** | 1220AA2100A |
| **TJ574** | DA10020BD0A | **TJ613** | 3D20032A000 | **TJ652** | 2120D33AA00 | **TJ691** | B2202A22001 | **TJ730** | D210DD1200A |
| **TJ575** | DD20D3AD10A | **TJ614** | 0A200002001 | **TJ653** | 2120DA3320A | **TJ692** | B34022AD30A | **TJ731** | AA102D2100A |
| **TJ576** | D210D20D200 | **TJ615** | 2DA0020AA0A | **TJ654** | 0A20DD20D01 | **TJ693** | 20200DA000A | **TJ732** | A22022D200A |
| **TJ577** | 31300330201 | **TJ616** | 3B20A2BB10A | **TJ655** | AD202A1020A | **TJ694** | 0430002000A | **TJ733** | AA202D3D00A |
| **TJ578** | B210220220A | **TJ617** | D2A0020AA0A | **TJ656** | 0F10212020A | **TJ695** | 2330D23020A | **TJ734** | 2A221DAAOOA |
| **TJ579** | 0120AB0A10B | **TJ618** | D2100B0D10A | **TJ657** | 0200243B20A | **TJ696** | 02101D3B200 | **TJ735** | 2222132D00A |
| **TJ580** | 2D20022220A | **TJ619** | 0A200A2220A | **TJ658** | 220000A320A | **TJ697** | 2200A03220A | **TJ736** | 2220A03D001 |
| **TJ581** | D120220A00A | **TJ620** | 22200A0A00A | **TJ659** | 02000432A00 | **TJ698** | 213023B2201 | **TJ737** | 2A10032D00A |
| **TJ582** | F110DA0220A | **TJ621** | 0230AB0D00A | **TJ660** | A100333000A | **TJ699** | 3B3042D2A00 | **TJ738** | 2A101322001 |
| **TJ583** | F2A0AD0320B | **TJ622** | 22202213A0A | **TJ661** | 01203230D0A | **TJ700** | 2300DD0DD0B | **TJ739** | 2DB2133200A |
| **TJ584** | 33A03D2DA0A | **TJ623** | 2220AEA200A | **TJ662** | 13202DD020A | **TJ701** | 23302D32A00 | **TJ740** | 1AB2A33200A |
| **TJ585** | 3320DA12001 | **TJ624** | E320230220A | **TJ663** | 03201A2320A | **TJ702** | 2D20DD32D0A | **TJ741** | AD20AA2200B |
| **TJ586** | E1200DADA0A | **TJ625** | CAA012A220A | **TJ664** | 0310122020A | **TJ703** | D320AD32A0A | **TJ742** | 0A202ABD00A |
| **TJ587** | B12012AD20A | **TJ626** | B230132D00A | **TJ665** | DD00201220A | **TJ704** | 2B203DA220A | **TJ743** | 22212D3A00A |
| **TJ588** | E110020220A | **TJ627** | 0230D32DA0B | **TJ666** | 123022A2201 | **TJ705** | 0A0032A320A | **TJ744** | 2D212222001 |
| **TJ589** | AA200F0D00A | **TJ628** | 3220032A20A | **TJ667** | 2120222210A | **TJ706** | 03203DB220A | **TJ745** | A13130A3001 |
| **TJ590** | A120030D00A | **TJ629** | 421002A220A | **TJ668** | 121032ABA0A | **TJ707** | 302024D2200 | **TJ746** | A1213DB200A |
| **TJ591** | 2D20042010A | **TJ630** | 2D20032A00B | **TJ669** | 2B20BA30D0A | **TJ708** | 002022B020A | **TJ747** | DA312F3200A |
| **TJ592** | 2220DD2A10A | **TJ631** | 3220132A00A | **TJ670** | 3330113020A | **TJ709** | 30302AB220A | **TJ748** | 2A3A2BBD00A |
| **TJ593** | B220AD0220A | **TJ632** | D2A0033D20A | **TJ671** | 01300320D0A | **TJ710** | 002024B0D0A | **TJ749** | 2A21D2A200A |
| **TJ594** | 02002410001 | **TJ633** | 21A00232002 | **TJ672** | 00201D3210A | **TJ711** | 0020DA2020A | **TJ750** | DD4022AD00A |
| **TJ751** | 2230DD2D00B | **TJ792** | 1A1130AA00A | **TJ833** | 02D000A210A | **TJ874** | 0A300230A0A | **TJ915** | 0D2020A200A |

| **TJ752** | AD302DAD00A | **TJ793** | 3B312D2200A | **TJ834** | 0D4000DDA0B | **TJ875** | 0DD0032210A | **TJ916** | 2A30A2AA00A |
| --- | --- | --- | --- | --- | --- | --- | --- | --- | --- |
| **TJ753** | 0A311B3200B | **TJ794** | D3111AAD00A | **TJ835** | 0AB00D3000A | **TJ876** | 031002D020A | **TJ917** | 2A202DA200A |
| **TJ754** | 023122AD00A | **TJ795** | 32211E2200A | **TJ836** | 02A0A2AD00B | **TJ877** | 0D200120201 | **TJ918** | AB301D3B00A |
| **TJ755** | 3131133D00D | **TJ796** | 230123BD00A | **TJ837** | 0A40B2A010A | **TJ878** | 0AA0013010A | **TJ919** | A1202032001 |
| **TJ756** | 2A21232200A | **TJ797** | 0A21222A00A | **TJ838** | 0230232020A | **TJ879** | 0A100320101 | **TJ920** | 12203022001 |
| **TJ757** | 2A11222B00B | **TJ798** | 21311DD100A | **TJ839** | 02A0232000A | **TJ880** | 0220A2D0A01 | **TJ921** | 3A202D2200A |
| **TJ758** | 0A21AA22000 | **TJ799** | 02A11D0200A | **TJ840** | 0A00433010B | **TJ881** | 2A302000001 | **TJ922** | 21201DAD00A |
| **TJ759** | 0D11A3BD001 | **TJ800** | 0321000D00A | **TJ841** | 0B3000A200A | **TJ882** | BAA03D3100A | **TJ923** | D020322000A |
| **TJ760** | AA3123A200A | **TJ801** | 0200020A20A | **TJ842** | 042002A100A | **TJ883** | 2A301421001 | **TJ924** | 20103A2D00A |
| **TJ761** | 0321122200A | **TJ802** | 0BA0022220A | **TJ843** | 0310032300A | **TJ884** | 2C10122D00A | **TJ925** | 1A3030AD00A |
| **TJ762** | 122AA423001 | **TJ803** | 0AB0A02010A | **TJ844** | 0AD0031210B | **TJ885** | DAB02132001 | **TJ926** | 1A00203A00A |
| **TJ763** | 0221D33000B | **TJ804** | 0D1022DD101 | **TJ845** | 0B20023A10A | **TJ886** | 0010213D00A | **TJ927** | A100323D001 |
| **TJ764** | D121A2A200A | **TJ805** | 0B000DAD201 | **TJ846** | 0330A231201 | **TJ887** | 3030302D00D | **TJ928** | BA10222200A |
| **TJ765** | AA31DA2D00B | **TJ806** | 0A30022210A | **TJ847** | 03D03DBA10A | **TJ888** | 30A0403000A | **TJ929** | D0201DB200A |
| **TJ766** | D2A1A3AD001 | **TJ807** | 02200023201 | **TJ848** | 0F00032010A | **TJ889** | 1A3022A300B | **TJ930** | 2C302FD200A |
| **TJ767** | 123132A200A | **TJ808** | 02000A0220A | **TJ849** | 02200D3D20A | **TJ890** | 2A2012A200A | **TJ931** | 2A202AD000A |
| **TJ768** | DDA1A32D00A | **TJ809** | 0A100AA3A0A | **TJ850** | 0D3002A120A | **TJ891** | 20D013AA00A | **TJ932** | 2A30A020001 |
| **TJ769** | 0D21232D00A | **TJ810** | 03A0003A20B | **TJ851** | 0DA0032220A | **TJ892** | 22304DA200A | **TJ933** | BA30D0B2001 |
| **TJ770** | 2A313B3200A | **TJ811** | 0200A03030A | **TJ852** | 03D0033D10A | **TJ893** | 3A301212001 | **TJ934** | 2B10232D00D |
| **TJ771** | 2221ABA000A | **TJ812** | 0DA002AB20A | **TJ853** | 0DE0022020A | **TJ894** | AA101A2200A | **TJ935** | 1A30123200A |
| **TJ772** | 2BA12A1A001 | **TJ813** | 01A0A2D210A | **TJ854** | 023003DD20A | **TJ895** | 2A202A2A00A | **TJ936** | 2A30D33300A |
| **TJ773** | 0A312DA200A | **TJ814** | 02A0D20210A | **TJ855** | 0AD00A3010A | **TJ896** | 303021AD00A | **TJ937** | 3A30123D00A |
| **TJ774** | AD21D12200D | **TJ815** | 0B2023DA101 | **TJ856** | 02B0002220A | **TJ897** | A020322D00A | **TJ938** | 2A10132000A |
| **TJ775** | BAA12B1100A | **TJ816** | 0A00B3D000A | **TJ857** | 0AA0003220A | **TJ898** | 2A202A2200A | **TJ939** | 1B20232000A |
| **TJ776** | A121223100B | **TJ817** | 0220D32220A | **TJ858** | 0230032020D | **TJ899** | 10303D2300A | **TJ940** | 2A20D42D00A |
| **TJ777** | 02A122AD00A | **TJ818** | 03202AA220B | **TJ859** | 0DA0022D10A | **TJ900** | AA30322200A | **TJ941** | 1A20102300A |
| **TJ778** | 22312BA2002 | **TJ819** | 0A002A20201 | **TJ860** | 01300330A01 | **TJ901** | D0D02B2D001 | **TJ942** | 31B0343300B |
| **TJ779** | AA3122BD00A | **TJ820** | 0130202D20A | **TJ861** | 0130D33010A | **TJ902** | 2130333200B | **TJ943** | 2130100300A |
| **TJ780** | 021132D200A | **TJ821** | 0220002020B | **TJ862** | 02A0A330201 | **TJ903** | ACA010A200A | **TJ944** | 2A102DB300A |
| **TJ781** | 03A124BD000 | **TJ822** | 0D20022220A | **TJ863** | 0BA0122020A | **TJ904** | DD1010BD00A | **TJ945** | 1D10302400A |
| **TJ782** | 2221222D000 | **TJ823** | 0310222220A | **TJ864** | 0120023230A | **TJ905** | D2A0AABA00A | **TJ946** | AD202FBD00A |
| **TJ783** | 2A31312D00B | **TJ824** | 0DD0AD2220A | **TJ865** | 0A30222320A | **TJ906** | AA10223200A | **TJ947** | 1D203FAA00A |
| **TJ784** | 2D2130AA001 | **TJ825** | OB302AA220A | **TJ866** | 04A0D2A020A | **TJ907** | 0A30202000B | **TJ948** | DA303D2200B |
| **TJ785** | 1221222D000 | **TJ826** | 0FA0A32D10A | **TJ867** | 02002233301 | **TJ908** | 1B20B02A00A | **TJ949** | AB20D0D200A |
| **TJ786** | 11311D2200A | **TJ827** | 030022B010A | **TJ868** | 0D300322A0A | **TJ909** | 2A201D2000A | **TJ950** | AD3013BD00A |
| **TJ787** | AAA12223001 | **TJ828** | 03D0022200A | **TJ869** | 01A002DD201 | **TJ910** | 2C20222000A | **TJ951** | 2D2023BA00A |
| **TJ788** | 13311DA2000 | **TJ829** | 0BA01220201 | **TJ870** | 0D00023010A | **TJ911** | AFA0A22200A | **TJ952** | BD20232D00B |
| **TJ789** | 0D1122BA00A | **TJ830** | 0A30A22220A | **TJ871** | 02A0A2A020A | **TJ912** | 2A302B2D00A | **TJ953** | AD2010AA00A |
| **TJ790** | 2D21323BOOA | **TJ831** | 0AD0223320A | **TJ872** | 0220033210A | **TJ913** | 2AA01DAA00A | **TJ954** | AD30203000B |
| **TJ791** | 3D1121A200A | **TJ832** | 0DA01DA020A | **TJ873** | 0240012210B | **TJ914** | D030A22D00B | **TJ955** | 2D20303200A |
