# Supplementary material for: Genetic Diversity of a Natural Population of Akebia trifoliata (Thunb.) Koidz and Extraction of a Core Collection Using Simple Sequence Repeat Markers
Source: Front Genet. 2021 Aug 31;12:716498. doi: 10.3389/fgene.2021.716498 (PMC8438410; doi:10.3389/fgene.2021.716498)
Supplement: Supplementary Table 3 — Na in core collection and random core collection at 28 SSR markers. [file Table_3.doc]

**Supplementary Table S3**

**N_a_ in core collection and random core collection at 28 SSR markers.**

| Marker | First random | Second random | Third random | Core collection |
| --- | --- | --- | --- | --- |
| s3 | 3 | 3 | 3 | 4 |
| s4 | 2 | 3 | 3 | 3 |
| s5 | 2 | 2 | 2 | 2 |
| s13 | 3 | 3 | 3 | 3 |
| s19 | 3 | 3 | 3 | 3 |
| s22 | 4 | 4 | 4 | 4 |
| s24 | 4 | 3 | 3 | 3 |
| s25 | 4 | 5 | 4 | 4 |
| s27 | 4 | 4 | 4 | 4 |
| s28 | 4 | 4 | 4 | 4 |
| s30 | 4 | 4 | 4 | 4 |
| s32 | 3 | 3 | 3 | 3 |
| s34 | 4 | 3 | 4 | 4 |
| s40 | 3 | 3 | 3 | 3 |
| s46 | 4 | 4 | 4 | 4 |
| s50 | 3 | 3 | 3 | 3 |
| s52 | 4 | 3 | 3 | 4 |
| s57 | 3 | 3 | 3 | 3 |
| s59 | 3 | 3 | 4 | 4 |
| s67 | 3 | 3 | 3 | 3 |
| s68 | 4 | 4 | 4 | 4 |
| s72 | 3 | 3 | 3 | 3 |
| s74 | 4 | 4 | 4 | 4 |
| s77 | 3 | 3 | 3 | 3 |
| s84 | 4 | 4 | 4 | 4 |
| s89 | 3 | 3 | 4 | 4 |
| s92 | 3 | 3 | 3 | 3 |
| s100 | 4 | 3 | 4 | 4 |
| mean | 3.3929 | 3.3214 | 3.4286 | 3.5000 |
