# Supplementary material for: Genetic Diversity of a Natural Population of Akebia trifoliata (Thunb.) Koidz and Extraction of a Core Collection Using Simple Sequence Repeat Markers
Source: Front Genet. 2021 Aug 31;12:716498. doi: 10.3389/fgene.2021.716498 (PMC8438410; doi:10.3389/fgene.2021.716498)
Supplement: Supplementary Table 4 — Ho in core collection and random core collection at 28 SSR markers. [file Table_4.doc]

**Supplementary Table S4**

**H_o_ in core collection and random core collection at 28 SSR marker**s.

| Marker | First random | Second random | Third random | Core collection |
| --- | --- | --- | --- | --- |
| s3 | 0.8506 | 0.8176 | 0.8056 | 0.7986 |
| s4 | 0.0000 | 0.0000 | 0.0000 | 0.0000 |
| s5 | 0.0000 | 0.0000 | 0.0000 | 0.0000 |
| s13 | 0.3506 | 0.3141 | 0.3822 | 0.3563 |
| s19 | 0.0490 | 0.0780 | 0.0403 | 0.0816 |
| s22 | 0.3313 | 0.4076 | 0.3416 | 0.3522 |
| s24 | 0.2368 | 0.2237 | 0.2405 | 0.2830 |
| s25 | 0.5166 | 0.4218 | 0.5294 | 0.4118 |
| s27 | 0.0892 | 0.1538 | 0.1316 | 0.1801 |
| s28 | 0.4714 | 0.4552 | 0.4786 | 0.4388 |
| s30 | 0.4932 | 0.4091 | 0.4379 | 0.3711 |
| s32 | 0.2089 | 0.1892 | 0.2119 | 0.2323 |
| s34 | 0.2403 | 0.3219 | 0.2416 | 0.2614 |
| s40 | 0.0600 | 0.1282 | 0.2368 | 0.1923 |
| s46 | 0.2480 | 0.2331 | 0.2541 | 0.2714 |
| s50 | 0.1063 | 0.0755 | 0.0982 | 0.0736 |
| s52 | 0.1946 | 0.2727 | 0.1795 | 0.2714 |
| s57 | 0.3425 | 0.3571 | 0.3404 | 0.3910 |
| s59 | 0.1026 | 0.1273 | 0.1074 | 0.1081 |
| s67 | 0.0764 | 0.0867 | 0.0738 | 0.0612 |
| s68 | 0.2252 | 0.3107 | 0.2632 | 0.2301 |
| s72 | 0.2540 | 0.3298 | 0.3068 | 0.2574 |
| s74 | 0.2500 | 0.2353 | 0.2143 | 0.2121 |
| s77 | 0.0000 | 0.0000 | 0.0476 | 0.0000 |
| s84 | 0.1649 | 0.0960 | 0.1863 | 0.1721 |
| s89 | 0.2712 | 0.2333 | 0.2564 | 0.2368 |
| s92 | 0.0311 | 0.0127 | 0.0123 | 0.0311 |
| s100 | 0.3684 | 0.3562 | 0.2986 | 0.3067 |
| mean | 0.2333 | 0.2374 | 0.2399 | 0.2351 |
