# Supplementary material for: Genetic Diversity of a Natural Population of Akebia trifoliata (Thunb.) Koidz and Extraction of a Core Collection Using Simple Sequence Repeat Markers
Source: Front Genet. 2021 Aug 31;12:716498. doi: 10.3389/fgene.2021.716498 (PMC8438410; doi:10.3389/fgene.2021.716498)
Supplement: Supplementary Table 5 — Nei’s in core collection and random core collection at 28 SSR markers. [file Table_5.doc]

**Supplementary Table S5**

**Nei’s in core collection and random core collection at 28 SSR markers.**

| Marker | First random | Second random | Third random | Core collection |
| --- | --- | --- | --- | --- |
| s3 | 0.5504 | 0.5493 | 0.5398 | 0.5591 |
| s4 | 0.1709 | 0.1976 | 0.1932 | 0.3906 |
| s5 | 0.0921 | 0.1284 | 0.2279 | 0.2248 |
| s13 | 0.5975 | 0.5759 | 0.6064 | 0.5971 |
| s19 | 0.1259 | 0.2894 | 0.2279 | 0.3975 |
| s22 | 0.4715 | 0.5096 | 0.4279 | 0.5449 |
| s24 | 0.3633 | 0.2940 | 0.3157 | 0.3840 |
| s25 | 0.6659 | 0.6835 | 0.6990 | 0.6663 |
| s27 | 0.3680 | 0.4542 | 0.3357 | 0.5457 |
| s28 | 0.6635 | 0.6278 | 0.6079 | 0.6779 |
| s30 | 0.6351 | 0.6117 | 0.6051 | 0.6677 |
| s32 | 0.2739 | 0.2318 | 0.2581 | 0.3480 |
| s34 | 0.3370 | 0.4570 | 0.4140 | 0.5094 |
| s40 | 0.5262 | 0.4862 | 0.4533 | 0.5627 |
| s46 | 0.3691 | 0.4377 | 0.4602 | 0.4933 |
| s50 | 0.3959 | 0.3263 | 0.2866 | 0.4601 |
| s52 | 0.4550 | 0.4664 | 0.4793 | 0.5739 |
| s57 | 0.3726 | 0.4258 | 0.3787 | 0.4764 |
| s59 | 0.2977 | 0.2937 | 0.2991 | 0.5139 |
| s67 | 0.4862 | 0.5162 | 0.5246 | 0.6038 |
| s68 | 0.6329 | 0.6458 | 0.6250 | 0.6355 |
| s72 | 0.5261 | 0.5418 | 0.5080 | 0.5939 |
| s74 | 0.6465 | 0.6635 | 0.6702 | 0.6752 |
| s77 | 0.5150 | 0.6235 | 0.6451 | 0.5799 |
| s84 | 0.3767 | 0.4355 | 0.3824 | 0.5211 |
| s89 | 0.5213 | 0.6044 | 0.5710 | 0.6859 |
| s92 | 0.4899 | 0.4880 | 0.4817 | 0.5258 |
| s100 | 0.4951 | 0.5154 | 0.4441 | 0.5818 |
| mean | 0.4436 | 0.4672 | 0.4524 | 0.5356 |
