# Supplementary material for: Genetic Diversity of a Natural Population of Akebia trifoliata (Thunb.) Koidz and Extraction of a Core Collection Using Simple Sequence Repeat Markers
Source: Front Genet. 2021 Aug 31;12:716498. doi: 10.3389/fgene.2021.716498 (PMC8438410; doi:10.3389/fgene.2021.716498)
Supplement: Supplementary Table 6 — PIC in core collection and random core collection at 28 SSR markers. [file Table_6.doc]

**Supplementary Table S6**

**PIC in core collection and random core collection at 28 SSR markers.**

| Marker | First random | Second random | Third random | Core collection |
| --- | --- | --- | --- | --- |
| s3 | 0.4605 | 0.4633 | 0.4550 | 0.4798 |
| s4 | 0.1563 | 0.1843 | 0.1804 | 0.3235 |
| s5 | 0.0879 | 0.1202 | 0.2019 | 0.1995 |
| s13 | 0.5240 | 0.5065 | 0.5304 | 0.5230 |
| s19 | 0.1216 | 0.2669 | 0.2114 | 0.3603 |
| s22 | 0.4145 | 0.4423 | 0.3768 | 0.4886 |
| s24 | 0.3339 | 0.2680 | 0.2853 | 0.3516 |
| s25 | 0.6029 | 0.6203 | 0.6411 | 0.6010 |
| s27 | 0.3395 | 0.4091 | 0.3097 | 0.4905 |
| s28 | 0.6071 | 0.5713 | 0.5516 | 0.6214 |
| s30 | 0.5742 | 0.5321 | 0.5297 | 0.6054 |
| s32 | 0.2565 | 0.2190 | 0.2406 | 0.3196 |
| s34 | 0.3112 | 0.4111 | 0.3741 | 0.4512 |
| s40 | 0.4634 | 0.4368 | 0.3940 | 0.4904 |
| s46 | 0.3414 | 0.3993 | 0.4222 | 0.4455 |
| s50 | 0.3349 | 0.2806 | 0.2489 | 0.3829 |
| s52 | 0.4104 | 0.4175 | 0.4273 | 0.5115 |
| s57 | 0.3383 | 0.3843 | 0.3378 | 0.4277 |
| s59 | 0.2677 | 0.2637 | 0.2747 | 0.4427 |
| s67 | 0.4343 | 0.4596 | 0.4561 | 0.5336 |
| s68 | 0.5692 | 0.5848 | 0.5622 | 0.5795 |
| s72 | 0.4292 | 0.4699 | 0.4365 | 0.5160 |
| s74 | 0.5821 | 0.6020 | 0.6085 | 0.6153 |
| s77 | 0.4244 | 0.5443 | 0.5724 | 0.5020 |
| s84 | 0.3461 | 0.3985 | 0.3522 | 0.4781 |
| s89 | 0.4663 | 0.5355 | 0.5044 | 0.6277 |
| s92 | 0.3758 | 0.3860 | 0.3714 | 0.4190 |
| s100 | 0.4351 | 0.4527 | 0.3937 | 0.5200 |
| mean | 0.3932 | 0.4154 | 0.4018 | 0.4753 |
